# Nanoporous Silicon with Graphene-like Coating for Pseudocapacitor Application

**DOI:** 10.3390/nano12132191

**Published:** 2022-06-26

**Authors:** Daria M. Sedlovets, Anton P. Naumov, Victor I. Korotitsky, Vitaly V. Starkov

**Affiliations:** Institute of Microelectronics Technology and High-Purity Materials, Russian Academy of Science (IMT RAS), 6 Academician Ossipyan Str., Moscow District, Chernogolovka 142432, Russia; apnaumov@iptm.ru (A.P.N.); vk58@inbox.ru (V.I.K.); starka@iptm.ru (V.V.S.)

**Keywords:** porous silicon, graphene-like coating, CVD, pseudocapacitors, cyclic stability

## Abstract

This paper presents the results of studies of the nanoporous silicon structure, both with different pore depths (up to 180 μm) and with layers in which a graphene-like coating was synthesized on the inner surface of the pores. The nanoporous layers were characterized by SEM as well as IR and Raman spectroscopy. Cyclic voltammetry and galvanostatic charge–discharge data in 3 M H_2_SO_4_ are presented as well as the results of the cyclic stability of these characteristics for the nanoporous structure. It was found that the degree of electrolyte pre-impregnation significantly affected the electrochemical processes, and the capacitance values depended on the depth (thickness) of the nanoporous layer. Increasing the thickness of the porous layer led to an increase in area-normalized pseudocapacity and was limited only by the mechanical strength of the structure. Performance improvement was also achieved by synthesis of the graphene-like layer in the volume of the nanoporous structure. The electrodes (composite materials) proposed in the work showed one of the best capacitive characteristics (87 mF/cm^2^ with 100% capacity retention after 15,000 cycles) in comparison with the data reported in the literature at present.

## 1. Introduction

Silicon is a common semiconductor material with the most advanced application technology. Since Si plates are the main platform for the integration of modern electronic devices, intensive research is aimed at finding potential applications of this material. Porous silicon (por-Si) is used to construct in-chip devices and has unique properties [1,2], which determine many of its applications in the following: sensors [3], molecular separators [4,5], micro fuel cells [6], Li-ion batteries [7], hydrogen storage [8,9], as well as electrochemical capacitors [10,11,12,13,14,15,16,17,18,19,20]. Among the afore-mentioned devices, ultracapacitors and pseudocapacitors are primarily distinguished, in which energy is either stored and released through electrostatic interactions between ions in the electrolyte and electrodes (the so-called ‘electrical double layer’), or is based on redox reaction near the surface of an active material, respectively.

The first attempts to use porous silicon as a capacitor electrode [10,11,12] led to insignificant results: the capacitances achieved were only on the order of hundreds μF/cm^2^. However, these values have since been greatly improved (from units to hundreds mF/cm^2^) by the authors [13,14,15,16,17,18,19,20]. An interesting challenge is the deposition of various coatings on porous silicon structures. For example, it has been proposed to decorate por-Si with metals (thin Au film [10] or Pd nanoparticles [21]) as well as with a TiN layer [13,17]. The most popular coatings are carbon-based materials: carbon sheath [14,15], nanocrystalline diamond [11], graphene oxide [21] and CVD-grown graphene [16,18,20,22]. Mainly, carbon coatings are either deposited on the surface [11], or used to cover shallow pores, up to 10 μm depth [16,18,20,22]. Successful results have been achieved by the authors [14,15] after carbon covering 80 μm and 120 μm por-Si layers to the full depth.

In this work, we determine how the graphene-like coating (GLC) synthesized on the inner surface of the Si nanopores (up to 150 μm thick) affects the electrochemical properties of por-Si. We will use the term ‘graphene-like’ hereafter to denote the material of well-defined graphene structure with the crystallite size of a few tens of nanometers. Based on the analysis of cyclic voltammograms, the features of redox processes that determine the pseudocapacitive characteristics are discussed. Capacitances of obtained composite material (por-Si + GLC) are compared with those of uncoated porous electrodes. Alterations to cyclic stability of modified electrodes are considered. We also compare the characteristics of nanoporous structures of various depths and analyze the literature data on por-Si structures used as capacitor electrodes.

## 2. Materials and Methods

### 2.1. Nanoporous Layer Formation

Single-crystal silicon plates with a diameter of 100 mm, a thickness of 460 μm and surface orientation (100) were used as wafers: the antimony-doped n-type Si (ρ_v_ = 0.008 Ω∙cm).

Silicon plates were cut into 12 × 18 mm^2^ samples using a sapphire scrubber. The samples were weighed using an HR-100AG Mettler Toledo balance (0.1 mg precision) before, and after, anodic etching. The polished surface of Si plates was subjected to anodic etching in standard conditions [1,23]. The process was carried out in galvanostatic mode at room temperature; the etching current density was selected in the range 80–100 mA/cm^2^. Etching was carried out in a solution of hydrofluoric acid and isopropanol (HF (49%): i-C_3_H_7_OH (98%) = 1:1). To increase wettability, 10^−3^ M cetyltrimethylammonium chloride (CH_3_(CH_2_)_15_N(CH_3_)_3_Cl) was added to the solution as a surfactant. The selected etching modes made it possible to form nanoporous layers with a columnar pore structure along the normal to the Si surface throughout the depth [23]. The required pore depth was achieved by selecting the appropriate time of anodic etching.

### 2.2. GLC Deposition

CVD of GLC was carried out through high-temperature (950 °C) low-pressure (∼100 Pa) pyrolysis of ethanol vapor in a carrier gas flow (Ar 99.999%). A detailed description of the synthesis process is given in Section S1 of Supporting Materials (SMs).

### 2.3. Characterization and Measurements

Raman spectra were taken with a Senterra micro-Raman system (Bruker, Berlin, Germany) in the 400–3700 cm^−1^ range resolution using a 532 nm laser.

Infrared (IR) spectra were obtained with an Hyperion 2000 IR microscope (Bruker, Berlin, Germany) using attenuated total reflection (ATR) objective with Ge crystal. 

Electrochemical measurements were performed on a computer-controlled potentiostat P-20X with a three-electrode cell E-7SF (Electrochemical Instruments, Chernogolovka, Russia) in a 3 M H_2_SO_4_. A porous silicon layer was used as the working electrode (electrical contact was performed through the silicon substrate), while an Ag/AgCl (saturated KCl-filled) electrode acted as reference and a graphite rod as counter electrode. The cell design ensured a strict constancy of the electrolyte-electrode contact area (0.785 cm^2^). The use of aqueous electrolytes limited the potential window in the range from −800 mV to 800 mV. Cyclic voltammograms (CVs) were recorded at the scan rates 100 mV/s, 50 mV/s, 20 mV/s, 10 mV/s and 5 mV/s. GCD measurements were performed at current density 1 mA/cm^2^. Electrochemical impedance spectra (EIS) were recorded in the 50 kHz–1 kHz interval.

For an undoubtedly accurate determination of the graphene coating effect on capacitance of por-Si, experiments were carried out as follows: electrochemical measurements were performed for uncoated por-Si, then the GLC was deposited into the pores of the same sample and measurements were repeated. Such a procedure allows the elimination of the influence of minor differences in depth and porosity of samples.

### 2.4. Calculations and Formulae

#### 2.4.1. CV Measurements

The specific capacitance was calculated by integrating the area under the CV curve to obtain the charge value and, then, dividing this by the mass of electroactive materials (or the surface area), the scan rate and the potential window, according to Equation (1) [15,24]:

(1) $$C=\frac{\int IdV}{\Delta V\cdot \vartheta \cdot m_{a}}=\frac{\int IdV}{\Delta V\cdot \vartheta \cdot A}$$

where *C* is the gravimetric or area-normalized capacitance in F/g or F/cm^2^, respectively, ∫*IdV* is the integrated area of the CV curve, Δ*V* is the scanned potential window in *V*, *ϑ* is the scan rate in V/s, m_a_ is the active mass of electrodes in g, A is the surface area which was exposed to electrolyte, in cm^2^.

#### 2.4.2. GCD Measurements

When measured in galvanostatic mode, specific capacitance was calculated from the GCD function as follows [15,24]:

(2) $$C=\frac{I_{c}\cdot \;\Delta t}{\Delta V\cdot m_{a}}=\frac{I_{c}\cdot \;\Delta t}{\Delta V\cdot A}$$

where *C* is the gravimetric or area-normalized capacitance in F/g or F/cm^2^, respectively, *I_c_* is the charging current in A, Δ*t* is the discharge time in s, Δ*V* is the scanned potential window in *V*, m_a_ is the active mass of electrodes in g, A is the surface area which was exposed to electrolyte, in cm^2^.

The hydrogen storage (HS) capacity was calculated using Equation (3) [21]:

(3) $$C_{s}=\frac{I_{c}\cdot \;\Delta t}{m_{a}}$$

where *C_s_* is the storage capacity in mAh/g, *I_c_* is the charging current in A, Δ*t* is the discharge time in h, m_a_ is the active mass of electrodes in g.

#### 2.4.3. Gravimetric Calculations

The active mass of electrode was calculated by subtracting the mass difference before, and after, etching from the mass of that volume of monocrystalline silicon plate which was subjected to the etching process (see Equation (4)). Porosity was estimated by dividing these values (see Equation (5)).

(4) $$m_{a}=V_{\mathrm{por}}\cdot \rho_{\mathrm{Si}}-\Delta m$$

(5) $$P=\frac{\Delta m}{V_{\mathrm{por}}\cdot \rho_{\mathrm{Si}}}\cdot 100\%$$

where m_a_ is the mass of porous layer, P is porosity, V_por_ is the volume of porous silicon, ρ_Si_ is the density of silicon plates, Δm is the difference mass before and after etching (the mass of silicon which was soluted during etching).

The calculation procedure is described in more detail in Section S2 from the SM document.

## 3. Results and Discussion

### 3.1. Sample Characterization

Porous silicon is a well-known material which has been studied in detail in many works (see review [1] and reference therein). Here, we briefly characterize the obtained structures. The applied modes of anodic etching [23] led to a nanocrystalline structure formation with vertical columnar pores [13,14].

**Figure 1:**
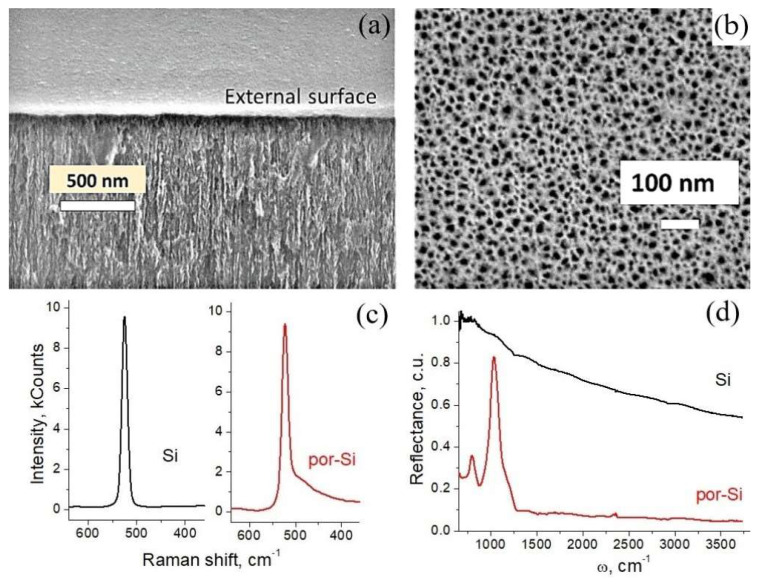
SEM images of por-Si layer: arbitrary cleavage (**a**) and top view (**b**). Raman (**c**) and IR (**d**) spectra of raw (black curve) and nanoporous (red curve) silicon.

Figure 1a shows a typical SEM image which is representative for near-surface porous layers with various pore depths. It should be noted that for the considered layers, morphologies along the pore depth were almost identical. So, the structure of the near-surface layer, shown in Figure 1a, was characteristic for the pore structure throughout the full depth for all the studied samples. A typical image of the outer surface of the porous layers is shown in Figure 1b (as can be seen, pore diameters are of 20 ± 10 nm).

Raman spectrum (Figure 1c) demonstrates that after the anodic etching, a silicon crystal Si band appeared at about 521 cm^−1^ without shifting; the peak also remained narrow and intense, which meant that the crystallinity was not disturbed [25]. An asymmetrical band distortion (shoulder on the right) was attributed to the transformation of an Si bulk crystal into a nanocrystalline structure of por-Si [26,27].

IR spectra in Figure 1d show that after etching, bonds with hydrogen and oxygen were formed: the peak at 804 cm^−1^ originated from twisting SiH_2_ vibrations; the broad band around 1050 cm^−1^ arose from stretching Si–O–Si vibrations [28]. An inevitable oxidation occurred during the etching process, as described in the literature [15,29]. The formation of SiH_2_ was also observed in the work [30]. Both of these functionalities could be involved in pseudocapacitive performance, while SiO_2_ could also influence the redox reaction kinetics, as will be discussed below.

Table 1 shows data on the etching depth and the calculated gravimetric porosity of the samples, depending on the etching time. Both values were almost proportional to the etching time.

**Figure 2:**
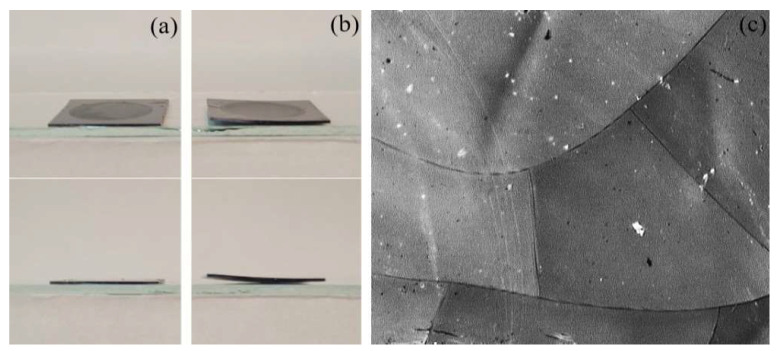
Photographs of 150 μm thick smooth sample (**a**) and 180 μm thick curved sample (**b**), as well as an optical microscope image of cracks on the surface of a 200 μm thick porous layer (**c**) after the impact of high temperatures (950 °C) and pressure drops (1–50 kPa) during GLC synthesis.

Importantly, an increase in depth of the porous layer was accompanied by a corresponding deterioration of the sample strength characteristics. Simultaneously, the porous layer/silicon substrate structure should withstand the impact of high temperatures (up to 950 °C) and pressure drops (1–50 kPa) during GLC synthesis on the inner surface of the pores. Figure 2 shows the effects of the mentioned factors on the structures with different pore depths.

The geometry and structure of samples with a porous layer thickness of 150 μm did not noticeably change (Figure 2a). The 180 μm thick layers were characterized by the deformation shown in Figure 2b. When the thickness of the porous layer was further increased up to 200 μm, cracking of the porous layer occurred (see Figure 2c).

An improvement in strength characteristics of the structures could be achieved by using previously developed technology for the formation of porous layers with “gradient porosity” in depth [31], or with a variable pore morphology along the layer thickness [32]. Such porous layers have increased strength and can be formed to a depth corresponding to almost the entire thickness of the silicon wafer (up to ~550 μm). However, the morphological features, as well as the considerable depth of gradient-porous structure require additional research on the applicability of these technologies to achieve increased capacitance.

When GLC was synthesized on the inner surface of the pores, the deposition time and the number of cycles of pressure drop were chosen so that the GLC covered the entire depth of por-Si structure. To make sure that this happened, the cleavage of the por-Si was studied using Raman spectroscopy: the spectra were taken at the near-surface, middle, and near-bottom points. As can be seen from Figure S4a–e (Section S4, SM), the GLC extended to the full depth of the structures. On the mentioned spectra, the second order 2D band was barely visible, since nanoporosity of silicon structures caused baseline distortion (maybe in consequence of overheating, due to strong absorption of laser light). D and G bands were wide and had almost the same intensity (this feature arose from the nanoscale lateral sizes of the graphene grains which constituted the GLC [33]). In the mentioned work, a TEM image of nanocrystalline GLC was also shown, which we reproduce in Figure S4f (SM).

### 3.2. Electrolyte Impregnation

As mentioned in the Introduction section, the charging process in pseudocapacitors is related to the redox reaction near the electrode surface. The prefix pseudo- is used to distinguish charge storage process from an electrical double layer mechanism in ultracapacitors. In our case, electrochemical charging arose from the faradic evolution of hydrogen [21,34]:

2H_3_O^+^ + 2e^−^ → H_2_ + 2H_2_O (6)

H_2_ − 2e^−^ → 2H^+^ (7)

In acidic medium water decomposition proceeds according to reaction:

2H_2_O ⇔ H_3_O^+^ + OH^−^ (8)

which is followed by the formation and decomposition of molecular hydrogen through the reaction 6 and 7, respectively.

The complex structure of the porous layer prevents the penetration of the electrolyte inside. Nanopores have an ultra-high aspect ratio (A_r_ ~4·10^3^ when pore height is about 80 μm and average diameter is about 20 nm) and the wetting of such structures is hindered. Simultaneously, with the most complete filling of pores with electrolyte, the increased surface contact (between electrolyte and electrode material) leads to the more active reactions occurring.

During the experiments, it was found that preliminary impregnation with the electrolyte strongly influenced the measurement results. Without pre-soaking in 3 M H_2_SO_4_ solution CV curves contained no obvious peaks attributed to chemical reactions. After preliminary exposure to the electrolyte, the capacitance increased significantly, due to the reversible redox process mainly observed at all scan rates: a reduction peak appeared in the cathodic direction between −0.2 V and −0.8 V, while an oxidation peak appeared in the anodic direction at 0.4 V and above (see SM, Section S5).

Thus, it was determined that the samples used in the experiment should be exposed to the electrolyte at room temperature for 7 or more hours before measurements. This time there was enough to ensure sufficient wettability of the electrolyte to the electrode and, therefore, to obtain accurate and maximized results. Boiling in 3 M H_2_SO_4_ solution was also used to enhance impregnation, but the results were practically the same compared to simple exposure at room temperature.

### 3.3. CV Measurements and EIS

**Figure 3:**
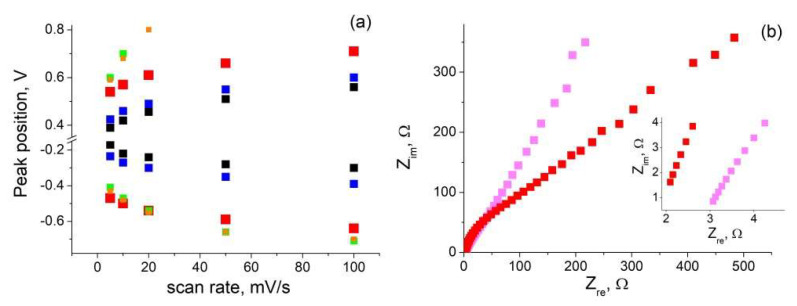
Dependence of the redox peak position on scan rate for por-Si samples of different depths: 13 μm (black), 19 μm (blue), 80 μm (red), 150 μm(green), 180 μm (orange). Numerical data are given in SM, Table S5a,b (**a**). Nyquist plots (inset: high frequency detail plots) for 80 μm sample before (pink) and after (red) GLC deposition (**b**).

After impregnation of samples with electrolyte, CVs were taken for por-Si with different depths of the porous layer before, and after, GLC synthesis (CVs shown in SM, Figure S5a–i). It was found that the position of the peaks depended on at least two factors. As seen from Figure 3a, redox peaks shifted towards higher voltage with increase in both scan rate and por-Si layer depth.

At increased scan rates, a rise in voltage at which redox processes occurred could be explained by (1) the kinetics of carrying out chemical reactions or (2) the carrier drift rate through an ultrathin dielectric layer (SiO_2_), which was inevitably formed in the por-Si structure during the etching process. For each scan speed, the oxidation peaks also shifted to the right with increasing por-Si depth (for 150 μm and 180 μm thick samples, they are outside the potential window). We attributed this to an increase in the SiO_2_ thickness with increasing etching time.

The same dependences of the peak positions were also characteristic for the GLC- coated sample. When comparing samples with, and without, GLC, the coated ones erre characterized by the redox peak shift in higher voltage. In the process of GLC synthesis, the already complex structure of por-Si was additionally modified and branched. As a result, the interaction with the electrolyte could be complicated, which increased the voltage required for the reaction to proceed. EIS measurements confirmed the presence of diffuse hindrances in the coated samples (see Figure 3b: the Nyquist plot for the composite material has a greater slope than that of the raw por-Si). The starting point in the high frequency region of the Nyquist plot (see inset in Figure 3b) corresponded to the resistance of the cell: 3Ω and 2Ω for the measurements of por-Si and por-Si + GLC electrodes, respectively. Therefore, the GLC-coated sample had less electrical resistance than uncoated por-Si, which was consistent with earlier studies [35]. Figure 3b shows the results of measurements for only one sample, but the described patterns were observed for all of them (see SM, Section S6).

Redox peak ‘intensity’ (maximal current) versus scan rate provided information about the process mechanism. Figure S5j,k in SM show a linear relationship between peak current and scan rate for 13–80 μm thick samples. These were attributed to surface faradaic processes [36]. Solution-based faradaic processes reflect in the square root relationship between peak current and scan rate [37], as observed for 150 μm thick samples (see Figure S5l, SM). This means that structures with a porous layer up to 80 μm depth are capacitive-type materials, while 150 μm thick sample exhibit battery-like behavior [38]. This interesting phenomenon was observed for the first time in this experiment (to the best of our knowledge, Si porous layers applied in electrochemical capacitors have been limited to 120 μm in depth to date).

**Figure 4:**
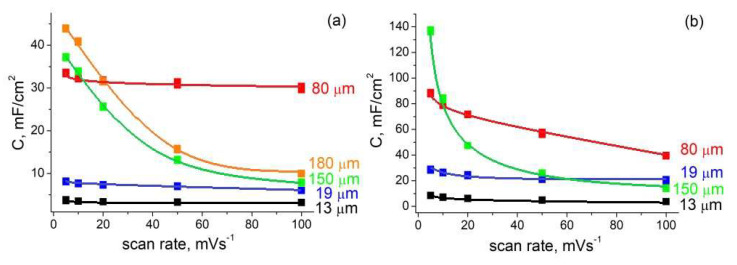
Scan rate dependence of the area-normalized capacitance at different depth of porous layer for the por-Si samples with (**b**) and without (**a**) GLC.

The capacitance values calculated from CV are summarized in Figure 4 (CV curves for the samples whose data are given here, are shown in Section S5, SM). It should also be noted that negligible capacitance was measured on non-etched silicon wafers (3 orders of magnitude less than on por-Si).

As seen in Figure 4, capacitance increased with increasing porous layer depth. Importantly, the increase in capacity at all scan rates was observed for samples with a pore depth of up to 80 μm. Deeper pores were also characterized by an increase in capacitance, but only at minimal scan rates (5–10 mV/s). Naturally, for 150 μm and 180 μm samples the capacitances decreased (at 20–100 mV/s) when an oxidation peak (the voltage at which the reaction 7 proceeds) was outside the potential window ([−800; 800] mV for aqueous electrolytes), because the charge of pseudocapacitors accumulates mainly during redox reactions.

The same depth dependencies of the capacitance values (growth with deepening of the pores) have been reported in [12,14], but in both works only area-normalized data were given. In this case, only an extensive increase in specific capacity occurred due to an increase in the working volume while the sample area exposed to the electrolyte remained the same. So, a qualitative capacity improvement needed to be confirmed by a simultaneous increase in gravimetric values. Here, we observed an increase in both the area-normalized (in mF/cm^2^) and gravimetric (in F/g) data for the samples with a pore depth of up to 80 μm (capacities per gram are shown in the Figure S7, SM). Such dependence could be explained by the different active surface areas of pore channels (not to be confused with the planar area exposed to the electrolyte). Deeper pores have larger surface area due to an increased porosity (in agreement with data from Table 1) and aspect ratio. An increased capacitance for materials with higher porosity was observed not only for por-Si [20], but also for other porous frameworks [39]. However, a further increase in the thickness of the porous layer (more than 80 μm) did not lead to intensive (qualitative) improvement in electrochemical capacity.

Since electrochemical measurements before and after synthesis were performed on the same samples, their characteristics could be directly compared. It could be seen that the capacitance increased significantly after GLC deposition (2–2.5 times). This improvement might be attributed to the increase in specific surface area after GLC deposition which was observed in the work [35]. The nanocrystalline structure of the GLC also matters: numerous graphene grain boundaries act as charge sites (reaction centers). The further increase in capacitance that can surely be achieved using organic electrolytes (due to the expansion of the potential window) is the subject of future research.

### 3.4. Cyclic Stability and GCD Measurements

**Figure 5:**
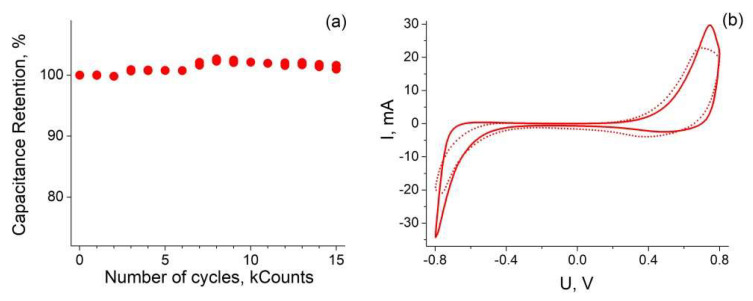
Dependence of capacity retention of graphene-coated 80 μm thick sample in 3 M H_2_SO_4_ from number of cycles (**a**). CV curves for the same sample at 100 mV/s scan rates before (solid curve) and after (dotted curve) cycling (**b**).

The GLC coated 80 μm thick sample showed excellent cyclic stability. There was no capacitance loss after 15,000 cycles at 100 mV/s in 3 M H_2_SO_4_ solution (see Figure 5). It could be seen that the capacitance even slightly increased, which was probably due to improvement in wettability. For example, in the work [20] 131% capacity retention after 10,000 cycles was observed.

GCD profiles for the 80 μm thick sample without GLC and for the same sample after GLC deposition are shown in SM (Figure S8a,b, respectively). The capacitances were calculated as equal to 18 mF/cm^2^ and 47 mF/cm^2^.

### 3.5. Comparison of Results

We tried to collect all the data regarding por-Si capacitors. Different electrolytes and various coatings were used in the works summarized in the Table 2, and their main similarity was the use of por-Si as electrodes for electrochemical capacitors. Based on the small thickness of the active layer in the mentioned papers (several microns or tens of microns, but not more than 120 μm), we considered only the area-normalized capacitance.

Notably, our maximum absolute result achieved for graphene-like-coated 150 μm thick sample was about 140 mF/cm^2^ at 5 mV/s scan rate, but we considered only the best intensive result for an 80 μm thick structure. Even so, our samples had superior characteristics, as compared to literature data, on por-Si electrochemical area-normalized capacitance in an aqueous electrolyte. The main reported values are as follows: units of mF/cm^2^ (~1 [11], 4.4 [17], 8 [20], 9 [13]) and less (0.2 [12] and 0.32 [10]). An amount of 26 mF/cm^2^ was achieved by Romanitan et al. [19] in 1M KCl through GLC deposition (however, 25% of this value was lost after just 1000 cycles). Ortaboy et al. measured 381 mF/cm^2^ [15] after deposition. Their sample not only had carbon coating, but also a manganese oxide layer with relatively poor cyclical stability (84% capacity retention after 5000 cycles). Close value (325 mF/cm^2^) with 83% capacity retention after 5000 cycles was reported by Alper et al. [14]. Importantly, the last two remarkable results (superior to our achievements) were obtained when measured in organic electrolyte (and, accordingly, in a wider potential window). Moreover, we demonstrated enhanced cyclic stability (complete capacity retention after 15,000 cycles). Such a long cyclic life is a great achievement for the pseudocapacitive application, since capacity loss is the weakness of this type of capacitor [40].

In the case of aqueous electrolyte use, hydrogen evolution reactions proceeded (see Section 3.2), so the electrodes proposed here could also be considered for application in hydrogen storage (HS) devices. Moreover, the 150 μm thick sample showed battery-type behavior (see Figure S5h, SM) and should be used as an accumulator. The HS capacity calculated from the GCD profile of this sample at low current density (see Figure S8c, SM) was about 20 mAh/g. There have been better values reported in the literature [21,30] during measurements in 3 M H_2_SO_4_, but the results we achieved demonstrate the ability to use our structures as HS devices. Targeted research in this direction could lead to improved HS performance.

## 4. Conclusions

It was observed that por-Si is sensitive to the degree of electrolyte impregnation when used as a pseudocapacitor electrode. The samples must be exposed to the electrolyte for several hours before measurements in order to obtain accurate and maximized results, because complete filling of pores with electrolyte provides for more active reactions.

It has been experimentally demonstrated that the thickness of the porous layer influences the electrochemical capacity of por-Si electrodes: deeper porous structures exhibit enhanced capacitance characteristics up to 80 μm (a further increase in pore depth leads to a change in the storage mechanism: 150 μm thick sample demonstrates battery-like behavior). An improvement has also been achieved through GLC deposition in the porous structure. The obtained composite materials were found to be perfect electrodes for electrochemical capacitors due to high and stable capacitive characteristics. Summarizing the literature data, the proposed method for modifying porous structures with a GLC allows the obtaining of superior characteristics, as compared to the electrochemical area-normalized capacitance of por-Si in an aqueous electrolyte reported by other authors. Graphene-coated samples also show excellent cyclic stability, as there was no loss in capacity after 15,000 cycles in 3 M H_2_SO_4_.

## Figures and Tables

**Table 1:** The depth of the porous layer and the calculated gravimetric porosity of samples that were etched for different times.

Etching time, min	3	5	20	45	60
Thickness of porous layer *, μm	13	19	80	150	180
Porosity, %	32	34	55	58	64

* determined from the images given in the SM, Section S3.

**Table 2:** The capacitance data obtained by different authors for por-Si with various coatings.

Ref.	Capacitance Achieved		Por-Si Characteristics		Electrolyte
	mF/cm^2^	at	Depth, m	Coating	
[15]	381	20 A/g	~ 80	carbon + MnO_x_	0.1 M EMIM-TSFI
[14]	325	1 mA/cm^2^	120	carbon	EMIM-TSFI
This work	47 87	1 mA/cm^2^ 5 mV/s	80	GLC	3 M H_2_SO_4_
[19]	26.31 ** 15.38 **	0.5 A/g 100 A/g	5	GLC	1 M KCl
[13]	9 **	–	6	TiN	0.1 M NaCl
[20]	8.16	1000 mV/s	–	few-layer graphene	
[17]	4.38 **	–	6	TiN	TEABF4/PC
[11]	0.99	100 mV/s	–	nanocryst. diamond	0.1 M KCl
[10]	0.32	–	43	Au	20% H_2_SO_4_
[12]	0.2	10 mV/s	–	–	0.25 M TEABF_4_/PC

** calculated from the data given in the corresponding papers (See details in SM, Section S9).

## Data Availability

Data available in a publicly accessible repository.

## Supplementary Materials

The following supporting information can be downloaded at: https://www.mdpi.com/article/10.3390/nano12132191/s1, Section S1. Graphene-like coating (GLC) synthesis. Section S2. Gravimetric calculation. Section S3. Thickness measurements. Section S4. Raman spectra of the composite structures (por-Si + GLC). Section S5. CV curves and their analysis. Section S6. EIS. Section S7. Gravimetric capacitance. Section S8. GCD data. Section S9. Area calculations from reported data.

## References

[1] Granitzer P., Rumpf K. (2010). Porous silicon—A versatile host material. Materials.

[2] Starkov V. (2009). Production, properties, and application of porous silicon. All Materials. Encycl. Ref. Book.

[3] RoyChaudhuri C. (2015). A review on porous silicon based electrochemical biosensors: Beyond surface area enhancement factor. Sens. Actuators B Chem..

[4] Lin X., Yang Q., Ding L., Su B. (2015). Ultrathin silica membranes with highly ordered and perpendicular nanochannels for precise and fast molecular separation. ACS Nano.

[5] Zhao Y., Gaur G., Retterer S.T., Laibinis P.E., Weiss S.M. (2016). Flow-through porous silicon membranes for real-time label-free biosensing. Anal. Chem..

[6] Presting H., Konle J., Starkov V., Vyatkin A., König U. (2004). Porous silicon for micro-sized fuel cell reformer units. Mater. Sci. Eng. B.

[7] Su X., Wu Q., Li J., Xiao X., Lott A., Lu W., Sheldon B.W., Wu J. (2014). Silicon-based nanomaterials for lithium-ion batteries: A review. Adv. Energy Mater..

[8] Lysenko V., Bidault F., Alekseev S., Zaitsev V., Barbier D., Turpin C., Geobaldo F., Rivolo P., Garrone E. (2005). Study of porous silicon nanostructures as hydrogen reservoirs. J. Phys. Chem. B.

[9] Shiraz H.G., Seyfollahi R. (2016). Hybrid system for potential room temperature hydrogen storage. Vacuum.

[10] Desplobain S., Gautier G., Semai J., Ventura L., Roy M. (2007). Investigations on porous silicon as electrode material in electrochemical capacitors. Phys. Status Solidi C.

[11] Ferreira N., Azevedo A., Beloto A., Amaral M., Almeida F., Oliveira F., Silva R. (2005). Nanodiamond films growth on porous silicon substrates for electrochemical applications. Diam. Relat. Mater..

[12] Rowlands S., Latham R., Schlindwein W. (1999). Supercapacitor devices using porous silicon electrodes. Ionics.

[13] Grigoras K., Keskinen J., Grönberg L., Yli-Rantala E., Laakso S., Välimäki H., Kauranen P., Ahopelto J., Prunnila M. (2016). Conformal titanium nitride in a porous silicon matrix: A nanomaterial for in-chip supercapacitors. Nano Energy.

[14] Alper J.P., Wang S., Rossi F., Salviati G., Yiu N., Carraro C., Maboudian R. (2014). Selective ultrathin carbon sheath on porous silicon nanowires: Materials for extremely high energy density planar micro-supercapacitors. Nano Lett..

[15] Ortaboy S., Alper J.P., Rossi F., Bertoni G., Salviati G., Carraro C., Maboudian R. (2017). MnO_x_-decorated carbonized porous silicon nanowire electrodes for high performance supercapacitors. Energy Environ. Sci..

[16] Chatterjee S., Carter R., Oakes L., Erwin W.R., Bardhan R., Pint C.L. (2014). Electrochemical and corrosion stability of nanostructured silicon by graphene coatings: Toward high power porous silicon supercapacitors. J. Phys. Chem. C.

[17] Grigoras K., Keskinen J., Grönberg L., Ahopelto J., Prunnila M. (2014). Coated porous Si for high performance on-chip supercapacitors. Journal of Physics: Conference Series.

[18] Oakes L., Westover A., Mares J.W., Chatterjee S., Erwin W.R., Bardhan R., Weiss S.M., Pint C.L. (2013). Surface engineered porous silicon for stable, high performance electrochemical supercapacitors. Sci. Rep..

[19] Romanitan C., Varasteanu P., Mihalache I., Culita D., Somacescu S., Pascu R., Tanasa E., Eremia S.A., Boldeiu A., Simion M. (2018). High-performance solid state supercapacitors assembling graphene interconnected networks in porous silicon electrode by electrochemical methods using 2, 6-dihydroxynaphthalen. Sci. Rep..

[20] Wu T.-H., Chang C.-T., Wang C.-C., Parwaiz S., Lai C.-C., Chen Y.-Z., Lu S.-Y., Chueh Y.-L. (2018). Few-layer graphene sheet-passivated porous silicon toward excellent electrochemical double-layer supercapacitor electrode. Nanoscale Res. Lett..

[21] Honarpazhouh Y., Astaraei F.R., Naderi H.R., Tavakoli O. (2016). Electrochemical hydrogen storage in Pd-coated porous silicon/graphene oxide. Int. J. Hydrogen Energy.

[22] Westover A.S., Freudiger D., Gani Z.S., Share K., Oakes L., Carter R.E., Pint C.L. (2015). On-chip high power porous silicon lithium ion batteries with stable capacity over 10000 cycles. Nanoscale.

[23] Zhang G.X. (2006). Porous silicon: Morphology and formation mechanisms. Modern Aspects of Electrochemistry.

[24] Lee J.W., Ko J.M., Kim J.-D. (2012). Hydrothermal preparation of nitrogen-doped graphene sheets via hexamethylenetetramine for application as supercapacitor electrodes. Electrochim. Acta.

[25] Münder H., Andrzejak C., Berger M., Klemradt U., Lüth H., Herino R., Ligeon M. (1992). A detailed Raman study of porous silicon. Thin Solid Film..

[26] Goodes S., Jenkins T., Beale M., Benjamin J., Pickering C. (1988). The characterisation of porous silicon by Raman spectroscopy. Semicond. Sci. Technol..

[27] Sui Z., Leong P.P., Herman I.P., Higashi G.S., Temkin H. (1992). Raman analysis of light-emitting porous silicon. Appl. Phys. Lett..

[28] Lenshin A., Kashkarov V., Seredin P., Spivak Y.M., Moshnikov V. (2011). XANES and IR spectroscopy study of the electronic structure and chemical composition of porous silicon on n-and p-type substrates. Semiconductors.

[29] Yang M., Shrestha N.K., Schmuki P. (2010). Toward self-ordered silica nanotubes by electrochemical anodization of Si (100). Electrochem. Solid State Lett..

[30] Merazga S., Cheriet A., M’hammedi K., Mefoued A., Gabouze N. (2019). Investigation of porous silicon thin films for electrochemical hydrogen storage. Int. J. Hydrogen Energy.

[31] Starkov V., Gavrilin E. (2007). Gradient-porous structure of silicon. Phys. Status Solidi C.

[32] Starkov V., Gosteva E., Sedlovets D., Kah M. (2018). Silicon structures with variable morphology of pores as a function of depth. Methods of obtaining physical and optical properties. J. Electrochem. Soc..

[33] Ferrari A.C. (2007). Raman spectroscopy of graphene and graphite: Disorder, electron-phonon coupling, doping and nonadiabatic effects. Solid State Commun..

[34] Jurewicz K., Frackowiak E., Béguin F. (2004). Towards the mechanism of electrochemical hydrogen storage in nanostructured carbon materials. Appl. Phys. A.

[35] Starkov V., Gosteva E., Sedlovets D., Belorus A. (2021). Nanoporous silicon structure with graphene coating. Microporous Mesoporous Mater..

[36] Pletcher D., Greff R., Peat R., Peter L., Robinson J. (2001). Instrumental Methods in Electrochemistry.

[37] Kissinger P.T., Heineman W.R. (1983). Cyclic voltammetry. J. Chem. Educ..

[38] Gogotsi Y., Penner R.M. (2018). Energy storage in nanomaterials–capacitive, pseudocapacitive, or battery-like?. ACS Nano.

[39] Lee D.Y., Shinde D.V., Kim E.-K., Lee W., Oh I.-W., Shrestha N.K., Lee J.K., Han S.-H. (2013). Supercapacitive property of metal–organic-frameworks with different pore dimensions and morphology. Microporous Mesoporous Mater..

[40] Zhao J., Burke A.F. (2021). Electrochemical capacitors: Materials, technologies and performance. Energy Storage Mater..

