# Supplementary material for: Nanoporous Silicon with Graphene-like Coating for Pseudocapacitor Application
Source: Nanomaterials (Basel). 2022 Jun 26;12(13):2191. doi: 10.3390/nano12132191 (PMC9268749; doi:10.3390/nano12132191)
Supplement: Supplementary file 1 [file nanomaterials-12-02191-s001.zip › nanomaterials-1786970-supplementary.pdf]

nanomaterials

MDPI

*Article*

# Nanoporous Silicon with Graphene-like Coating for Pseudocapacitor Application

**Daria M. Sedlovets *, Anton P. Naumov, Victor I. Korotitsky and Vitaly V. Starkov**

Institute of Microelectronics Technology and High-Purity Materials, Russian Academy of Science (IMT RAS), 6 Academician Ossipyan Str., Moscow District, Chernogolovka 142432, Russia; apnaumov@iptm.ru (A.P.N.); vk58@inbox.ru (V.I.K.); starka@iptm.ru (V.V.S.)

* Correspondence: sedlovets@iptm.ru; Tel.: +7-4965244190

## Section S1. Graphene-like coating (GLC) synthesis

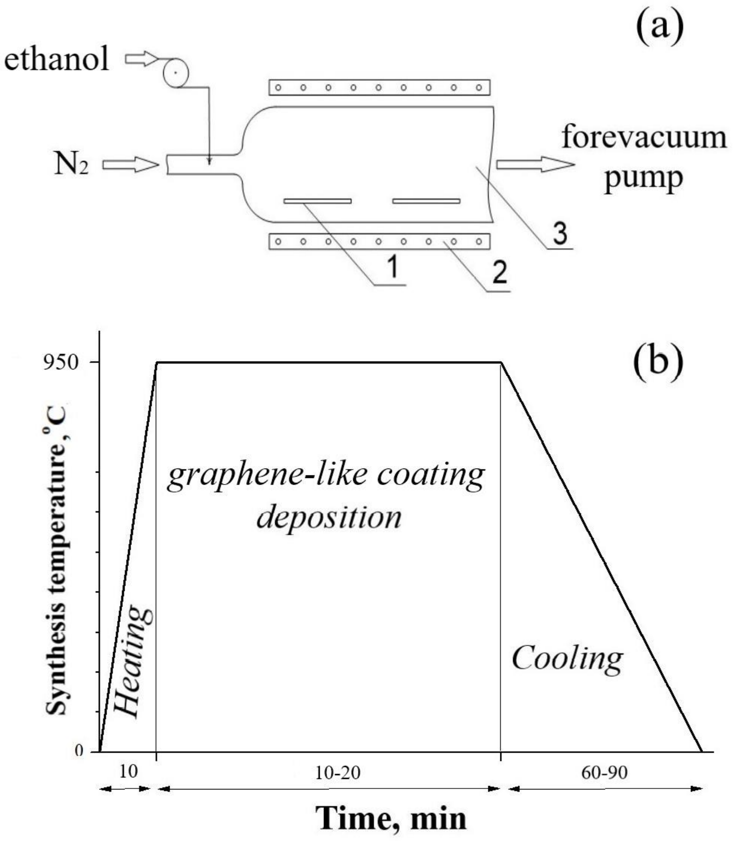


**Figure S1.** Schematic of the lab-made setup used to grow GLC from $C_2H_5OH$ vapor: 1 – substrates, 2 – external electric furnace, 3 – quartz reactor (**a**). The synthesis diagram (**b**).

GLC growth was carried out in a horizontal quartz reactor 1 (of 50 cm length and 3 cm inner diameter) incorporated in a tubular furnace 2 with a temperature control system: SPC-1-50 power unit (Autonics, South Korea) and TZN-4S controller (Autonics, Busan, South Korea) which heated the CVD set-up to a required temperature for 10 min. The inlet and outlet of the reactor were linked to the reagent supply system and forevacuum pump VI-2120 (Value, Wenling, China), respectively. The liquid ethanol from the graduated burette was fed through a peristaltic pump Sci Q400 (Watson

Marlow, Falmouth, UK) into the reactor where it was vaporized. The carrier gas flow was maintained with gas flow regulator RRG-10 (Eltochpribor, Moscow, Russia). The working pressure (1 kPa) was measured with a vacuum gauge DVR2 (Vacuubrand, Wertheim, Germany).

As described in (https://doi.org/10.1016/j.micromeso.2021.110981; 8 June 2022) for carbon coating deposition inside the porous structure (not only on the planar surface) sharp pressure drops (SPD) during synthesis are required. For this purpose, the reactor was serially filled with a precursor gas ($C_2H_5OH$ + Ar) to a pressure 50 kPa, and then the CVD system was quickly pumped out to the initial (working) pressure. The number of cycles during synthesis and deposition time determine the depth of carbon deposition in the pores.

**Section S2. Gravimetric calculation**

For each sample, the diameter (d) of porous layer was measured, and also, after electrochemical measurements, it was cleaved, and the depth (h) was determined.

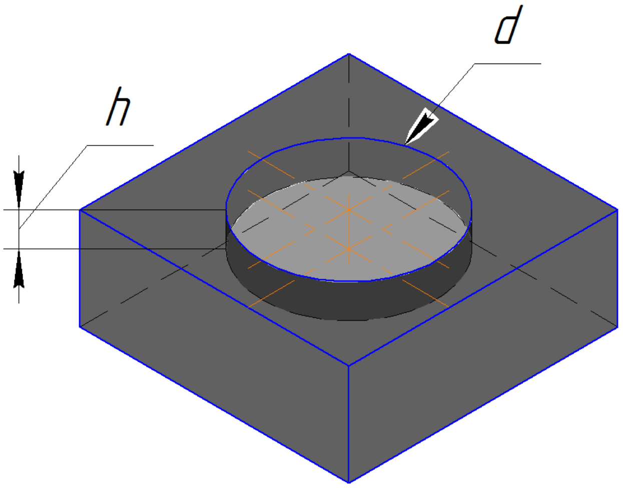


**Figure S2.** Schematic of monocystalline silicon sample with porous layer. Etched part enclosed in a cylindrical volume with height h and diameter d.

The volume (V) of cylindrical part which shown in Figure S2 was calculated from equation $V = (\pi d^2/4) \times h$.

The silicon wafer density (  was calculated as the ratio of unetched silicon wafer mass to its volume (2.2 g/cm$^3$).

A mass of that volume of monocrystalline silicon which was subjected to the etching process ($m_1$) was calculated from equation $m_1 = V \times p$.

The sample mass before ($m_2$) and after ($m_3$) etching was directly measured.

Since ($m_2 - m_3$) is a "mass of pores" (the mass of a silicon crystal that had dissolved during the etching), then the active mass ($m_a$) of por-Si and its porosity (P) are:

$m_a = m_1 - (m_2 - m_3)$;

$P = (m_1 - m_a)/m_1$.

**Section S3. Thickness measurements**

After electrochemical measurements, the samples were split and the thickness of the porous layer on the cleavage was directly measured using an optical microscope.

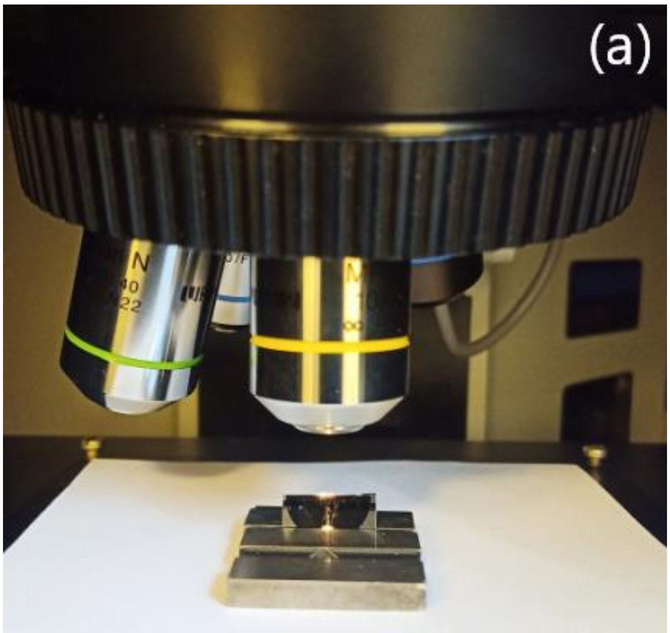


**Figure S3a.** A photograph of a spitted sample fixed in a magnetic holder.

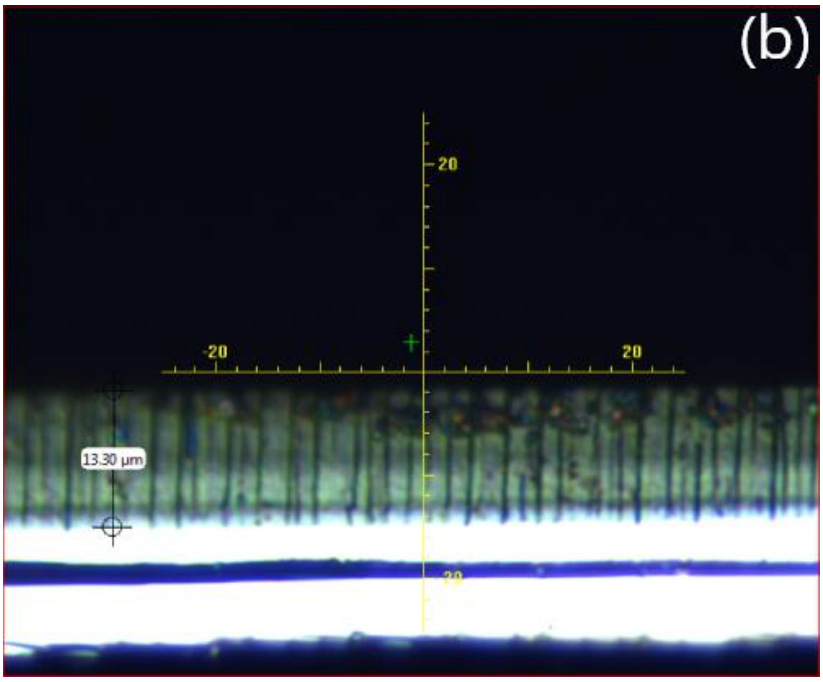


**Figure S3b.** An optical image of an arbitrary cleavage of the sample, which was etched during 3 min. Screen captured image was acquired using an optical microscope with 50x objective.

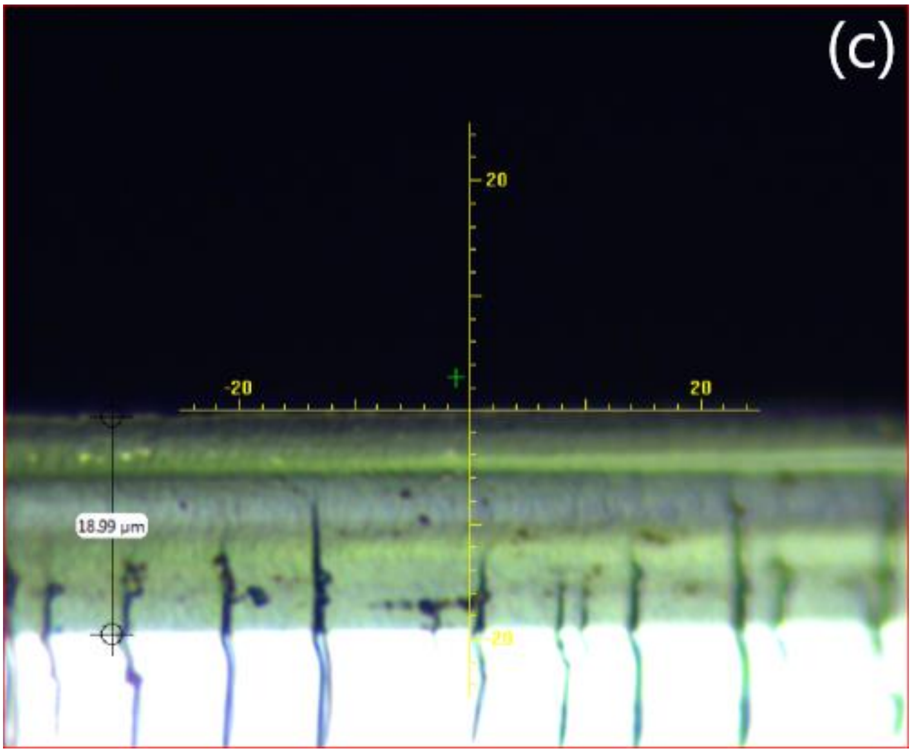


**Figure S3c.** An optical image of an arbitrary cleavage of the sample, which was etched during 5 min. Screen captured image was acquired using an optical microscope with 50x objective.

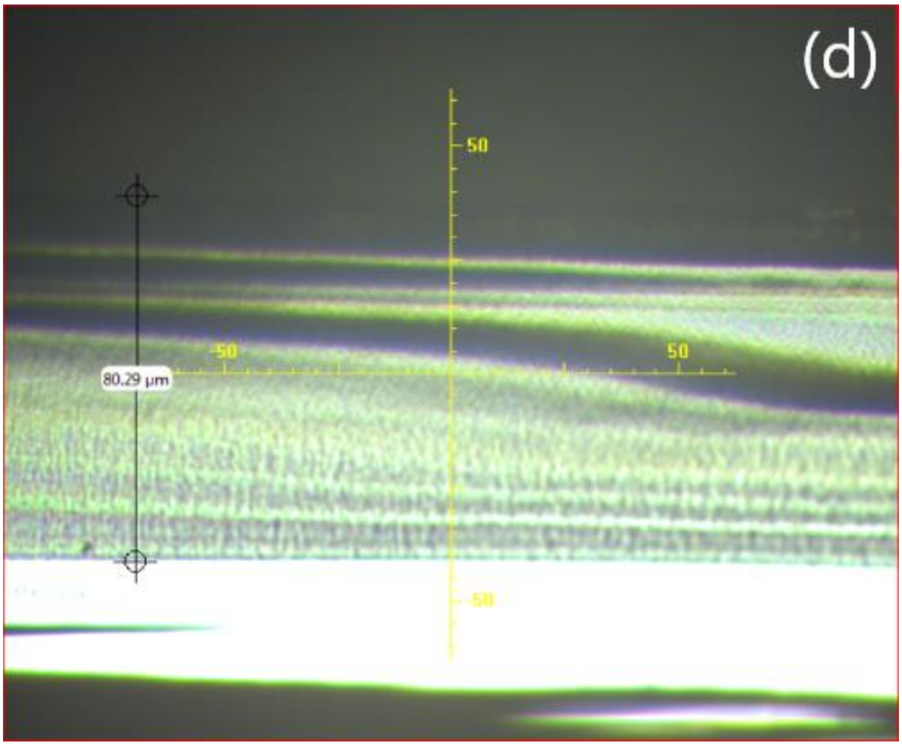


**Figure S3d.** An optical image of an arbitrary cleavage of the sample, which was etched during 20 min. Screen captured image was acquired using an optical microscope with 20x objective.

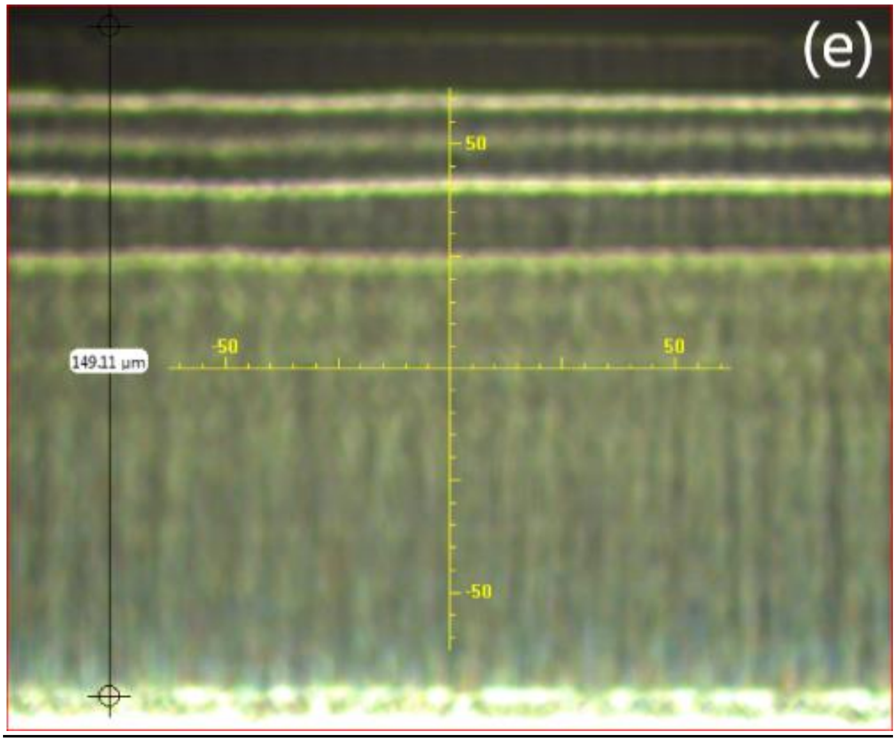


**Figure S3e.** An optical image of an arbitrary cleavage of the sample, which was etched during 45 min. Screen captured image was acquired using an optical microscope with 20x objective.

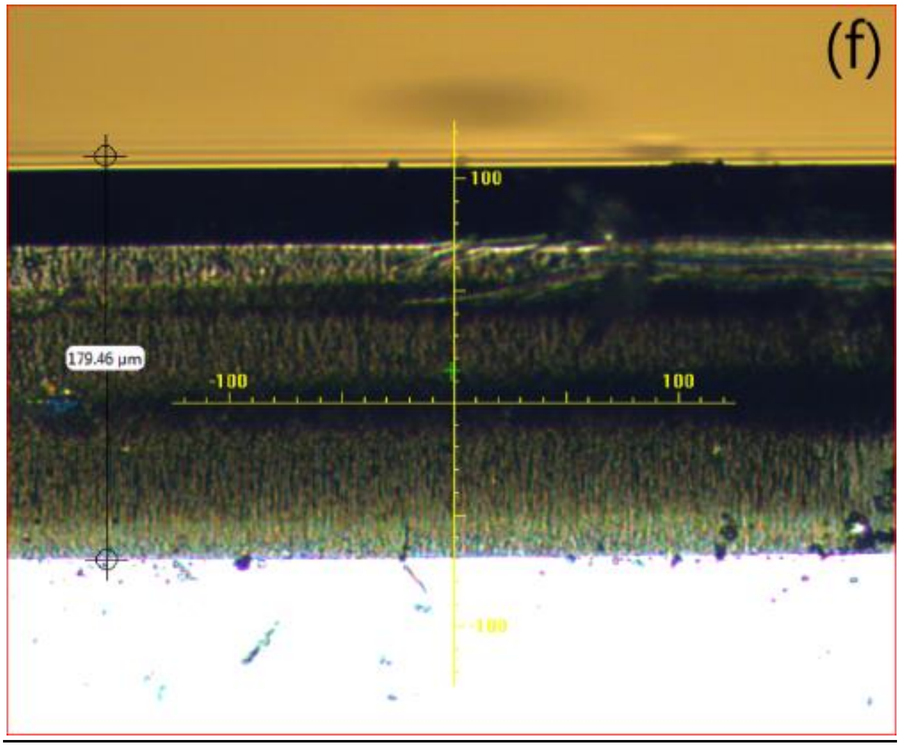


**Figure S3f.** An optical image of an arbitrary cleavage of the sample, which was etched during 60 min. Screen captured image was acquired using an optical microscope with 50x objective.

**Section S4. Raman spectra of the composite structures (por-Si + GLC)**

After electrochemical measurements the samples were split and studied using Raman spectroscopy. Below are the Raman spectra of the cleavage of different samples at various depths. Spectrum difference is explained by the fact that the method is very sensitive to focusing and, accordingly, strongly depends on the quality of the cleavage which cannot be reproducibly obtained.

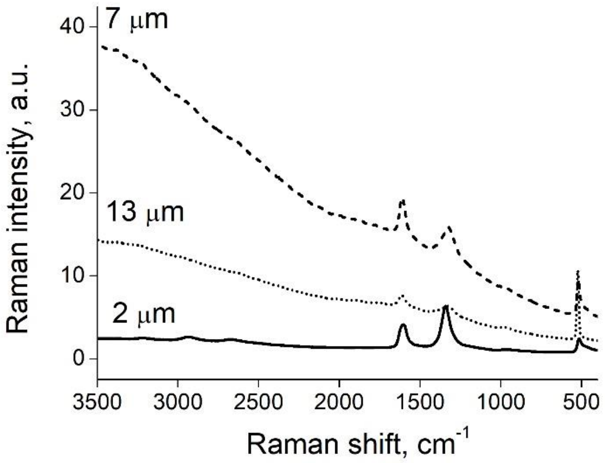


**Figure S4a**. 13 μm thick sample.

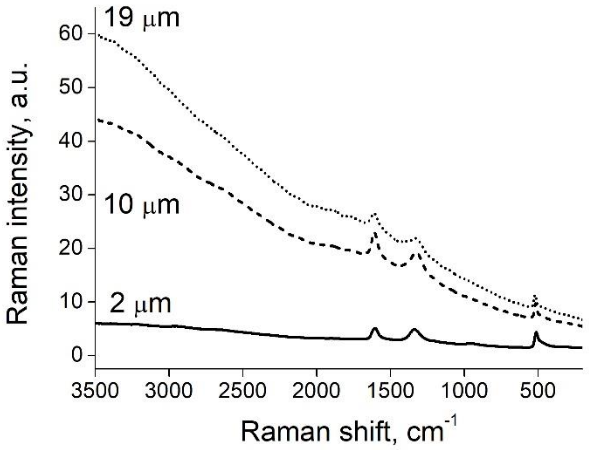


**Figure S4b**. 19 μm thick sample.

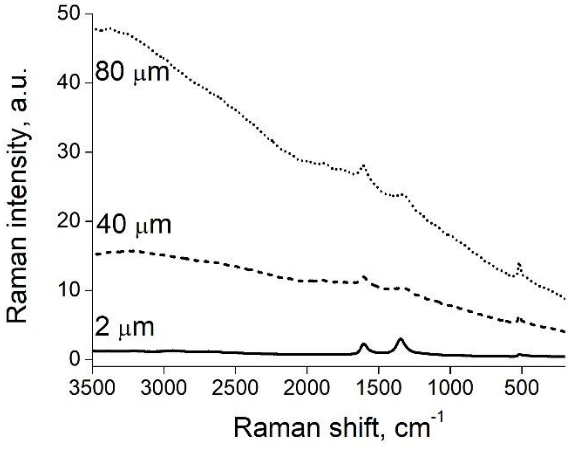


**Figure S4c**. 80 μm thick sample.

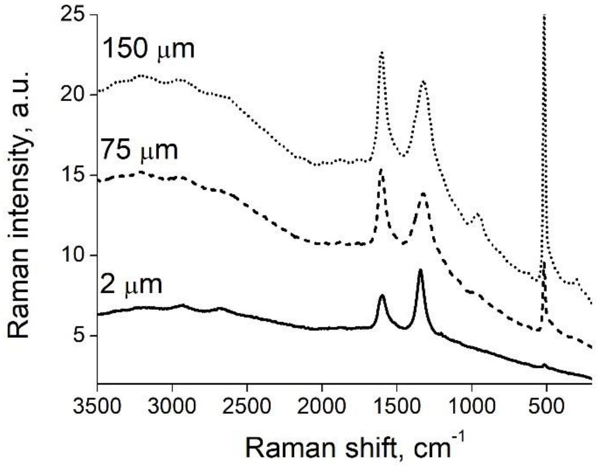


**Figure S4d**. 150 μm thick sample.

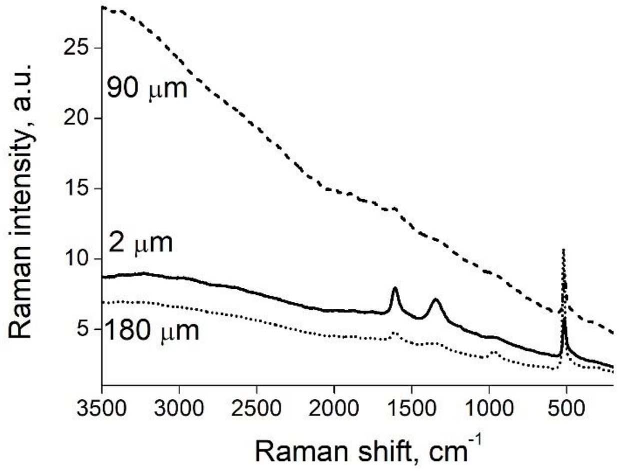


**Figure S4e**. 180 μm thick sample.

Electron diffraction shows a well-defined diffraction ring, a typical signature of nanocrystalline material.

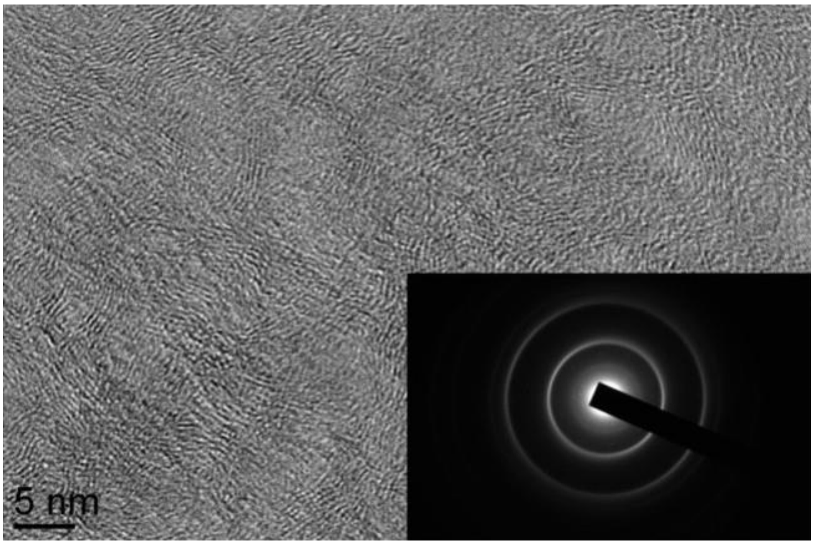


**Figure S4f**. TEM image of graphene-like film, synthesized on $SiO_2$/Si. Insert: electron diffraction. Reproduced with permission from (https://doi.org/10.1016/j.micromeso.2021.110981, 8 June 2022).

**Section S5. CV curves and their analysis**

CV curves for the samples with different depth of porous layer at various scan rates: 100 mV/s (red), 50 mV/s (blue), 20 mV/s (green), 10 mV/s (magenta) and 5 mV/s (black).

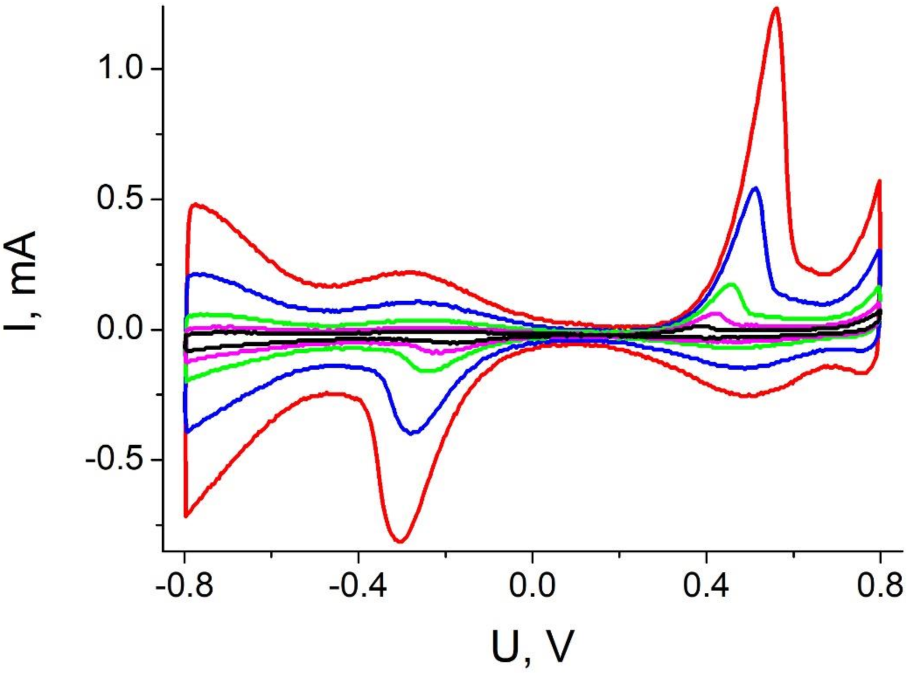


**Figure S5a**. 13 μm thick sample before GLC synthesis.

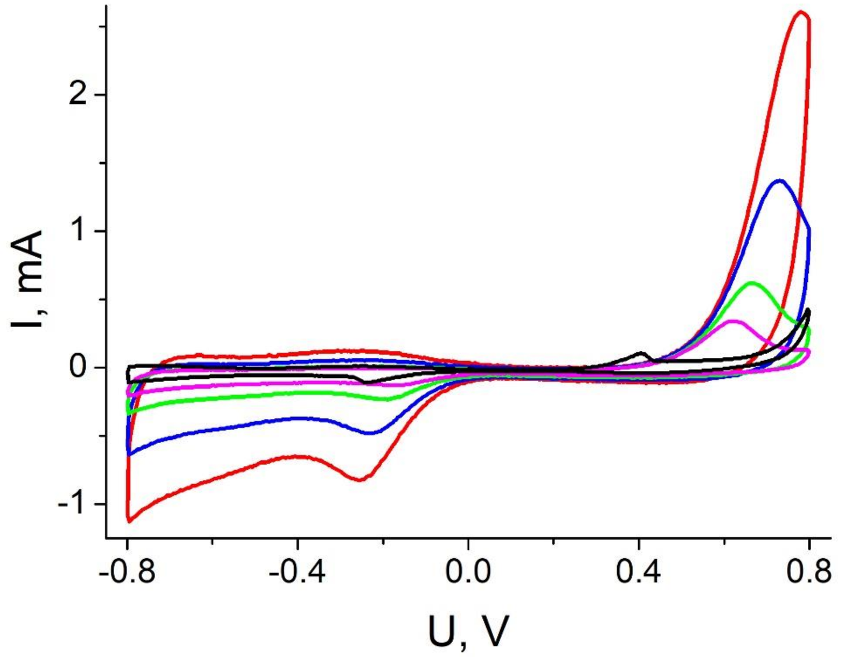


**Figure S5b**. 13 μm thick sample after GLC synthesis.

.

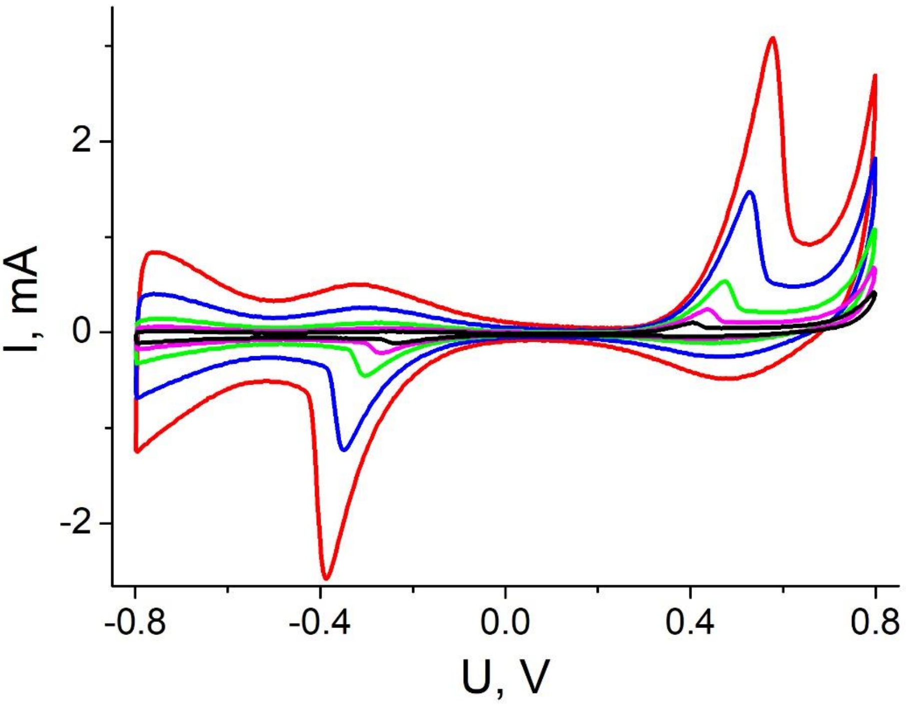


**Figure S5c**. 19 μm thick sample before GLC synthesis.

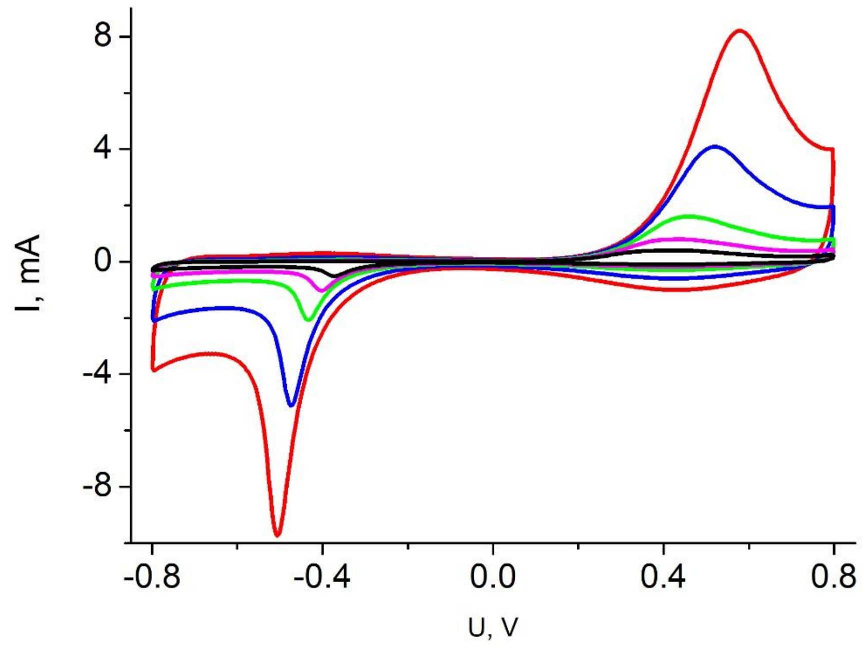


**Figure S5d**. 19 µm thick sample after GLC synthesis.

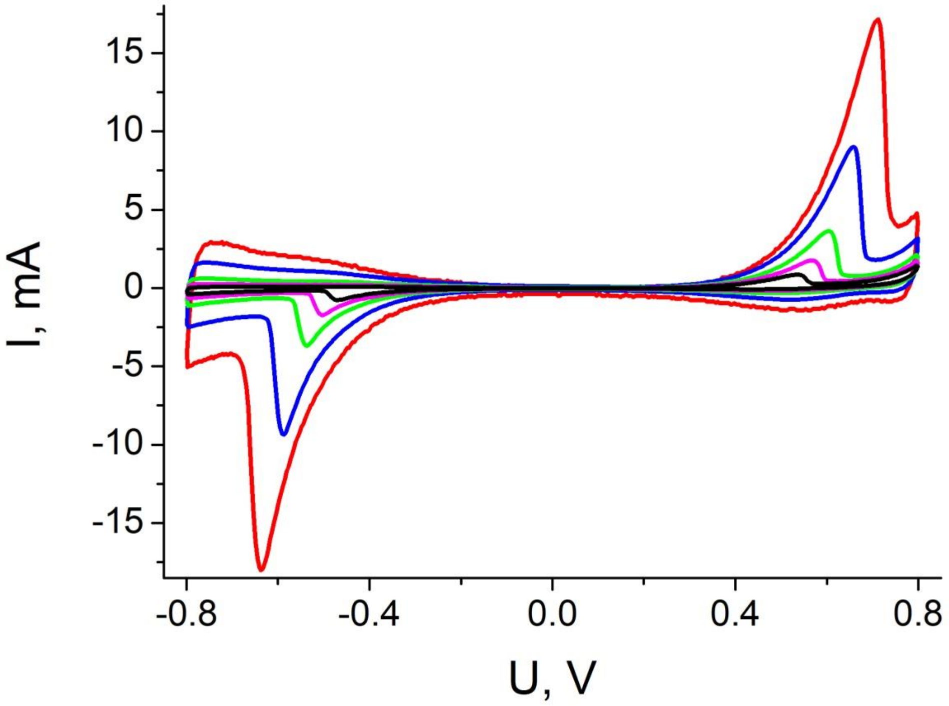


**Figure S5e**. 80 µm thick sample before GLC synthesis.

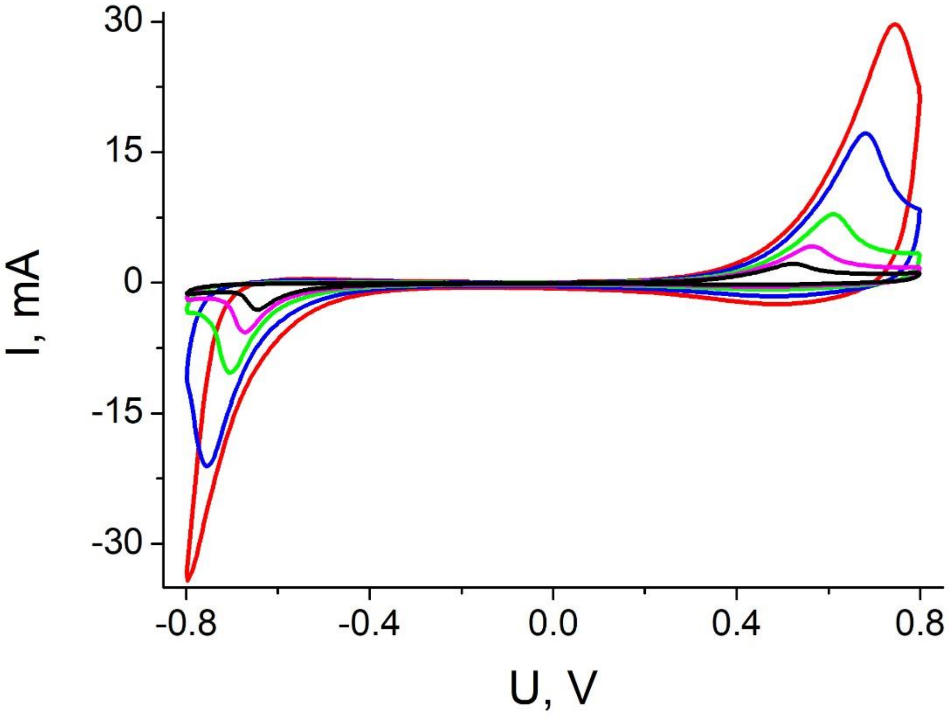


**Figure S5f**. 80 μm thick sample after GLC synthesis..

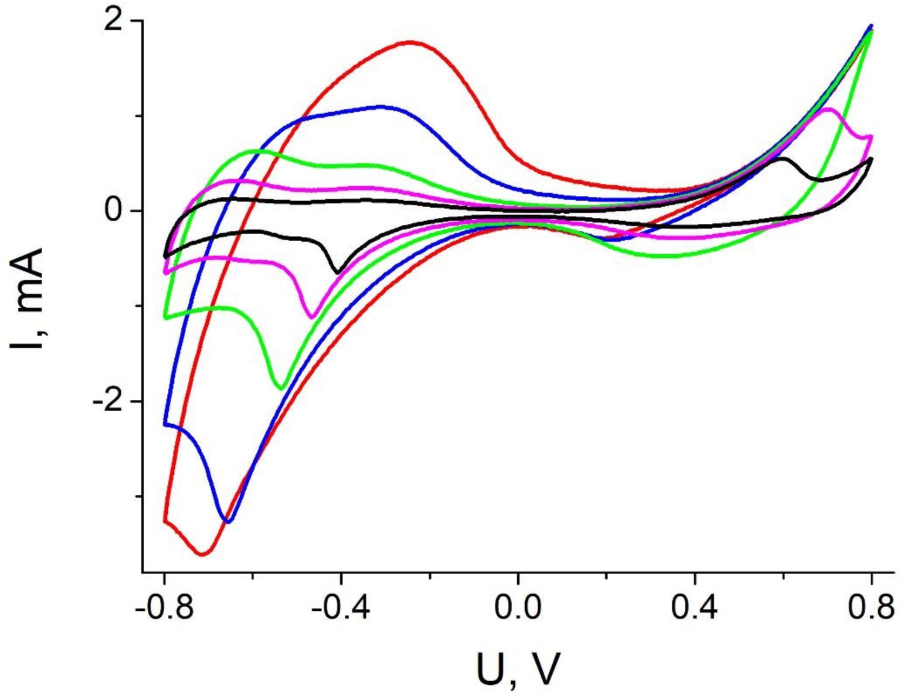


**Figure S5g**. 150 μm thick sample before GLC synthesis.

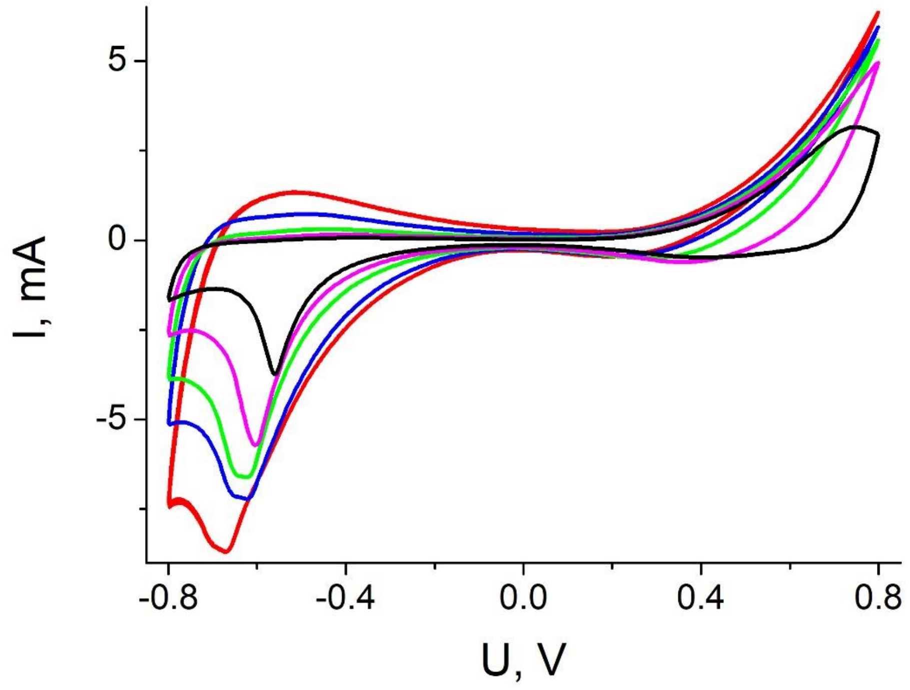


**Figure S5h**. 150 μm thick sample after GLC synthesis...

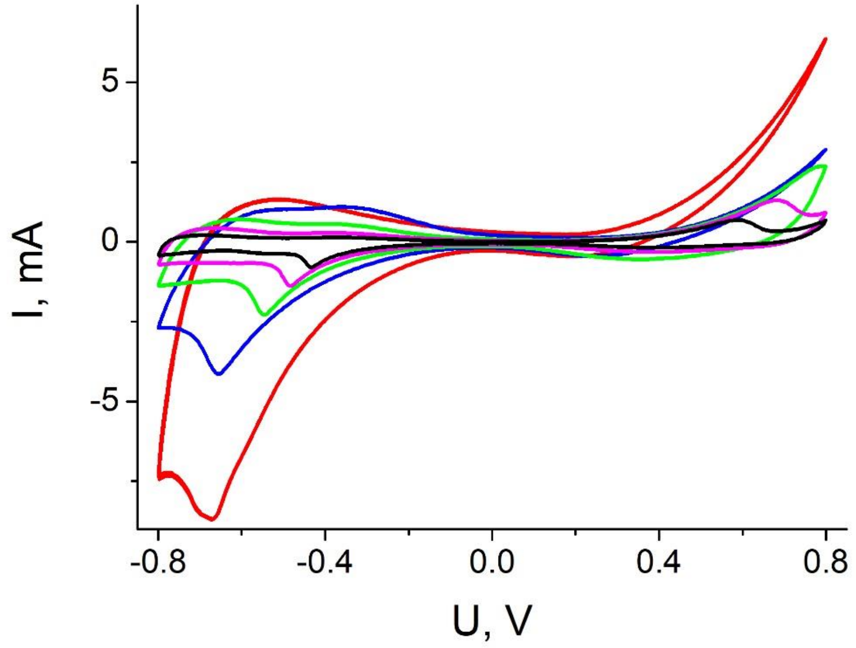


**Figure S5i**. 180 μm thick sample without GLC.

**Table S5a.** Peak position (voltage) values determined from the above CV curves for different samples at various scan rates.

| Peak position, V | | | | | | | | | | |
|---|---|---|---|---|---|---|---|---|---|---|
| | **5 mVs$^{-1}$** | | **10 mVs$^{-1}$** | | **20 mVs$^{-1}$** | | **50 mVs$^{-1}$** | | **100 mVs$^{-1}$** | |
| **13 µm** | -0.17 | +0.39 | -0.22 | +0.42 | -0.24 | +0.46 | -0.28 | +0.51 | -0.30 | +0.56 |
| **13 µm+ GLC** | -0.17 | +0.58 | -0.18 | +0.62 | -0.2 | +0.67 | -0.23 | +0.72 | -0.25 | +0.78 |
| **19 µm** | -0.24 | +0.43 | -0.27 | +0.46 | -0.30 | +0.49 | -0.35 | +0.55 | -0.39 | +0.60 |
| **19 µm+ GLC** | -0.37 | +0.42 | -0.40 | +0.43 | -0.43 | +0.46 | -0.47 | +0.52 | -0.5 | +0.58 |
| **80 µm** | -0.4 | +0.51 | -0.44 | +0.59 | -0.48 | +0.69 | -0.57 | o/r* | -0.65 | o/r |
| **80 µm+ GLC** | -0.65 | +0.52 | -0.67 | +0.56 | -0.7 | +0.61 | -0.76 | +0.68 | -0.8 | +0.74 |
| **150 µm** | -0.41 | +0.6 | -0.47 | +0.7 | -0.54 | o/r | -0.66 | o/r | -0.71 | o/r |
| **150 µm + GLC** | -0.56 | +0.74 | -0.6 | o/r | -0.63 | o/r | -0.63 | o/r | -0.68 | o/r |
| **180 µm** | -0.43 | +0.59 | -0.48 | +0.68 | -0.55 | +0.8 | -0.66 | o/r | -0.7 | o/r |

* o/r – out of range

**Table S5b.** Peak intensity (current) values determined from the above CV curves for different samples at various scan rates.

| Peak intensitiy, mAcm$^{-2}$ | | | | | | | | | | |
|---|---|---|---|---|---|---|---|---|---|---|
| | **5 mVs$^{-1}$** | | **10 mVs$^{-1}$** | | **20 mVs$^{-1}$** | | **50 mVs$^{-1}$** | | **100 mVs$^{-1}$** | |
| **13 µm** | -0.03 | +0.01 | -0.07 | +0.05 | -0.13 | +0.14 | -0.31 | +0.42 | -0.63 | +0.97 |
| **13 µm+ GLC** | -0.09 | +0.09 | -0.1 | +0.26 | -0.18 | +0.49 | -0.38 | +1.07 | -0.64 | +2.04 |
| **19 µm** | -0.08 | +0.08 | -0.17 | +0.18 | -0.37 | +0.42 | -1 | +1.2 | -2 | 2.4 |
| **19 µm+ GLC** | -0.4 | +0.36 | -0.78 | +0.65 | -1.6 | +1.27 | -4 | +3.2 | -7.7 | +6.5 |
| **80 µm** | -0.6 | +0.7 | -1.3 | +1.5 | _2.9 | +2.8 | -7.3 | +7.1 | -14.1 | +13.4 |
| **80 µm+ GLC** | -2.5 | +1.7 | -4.5 | +3.4 | -8.2 | +6.2 | -16.6 | +13.5 | -27 | +23.4 |
| **150 µm** | -0.5 | +0.43 | -0.87 | +0.84 | -1.45 | o/r | -2.55 | o/r* | -2.83 | o/r |
| **150 µm + GLC** | -2.94 | +2.48 | -4.5 | o/r | -5.2 | o/r | -5.6 | o/r | -6.8 | o/r |

* o/r – out of range

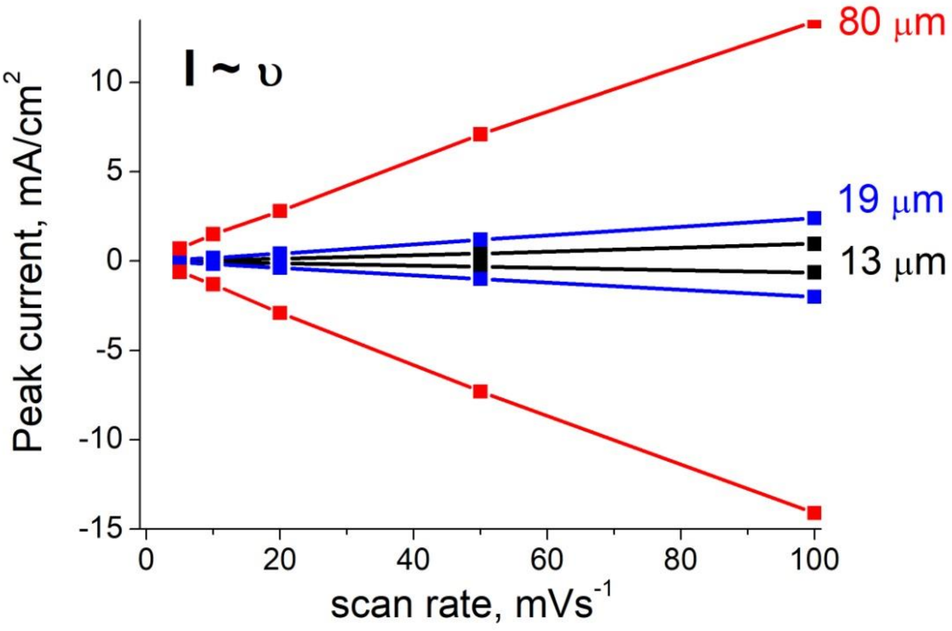


**Figure S5j.** Capacitive-type (linear) scan rate dependence of the peak current at different depth of porous layer (13 μm – black; 19 μm – blue; 80 μm – red) for the por-Si samples without GLC.

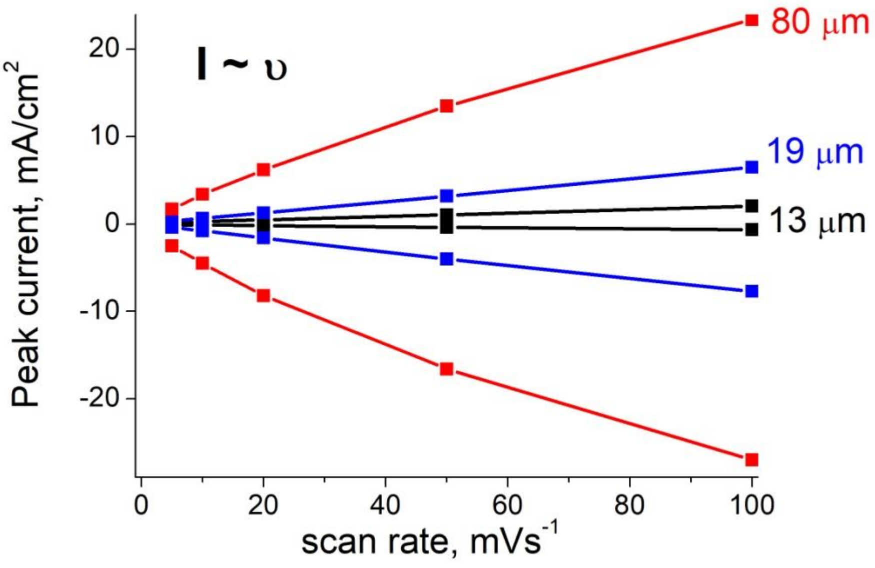


**Figure S5k.** Capacitive-type (very close to linear) scan rate dependence of the peak current at different depth of porous layer (13 μm – black; 19 μm – blue; 80 μm – red) for the por-Si samples with GLC.

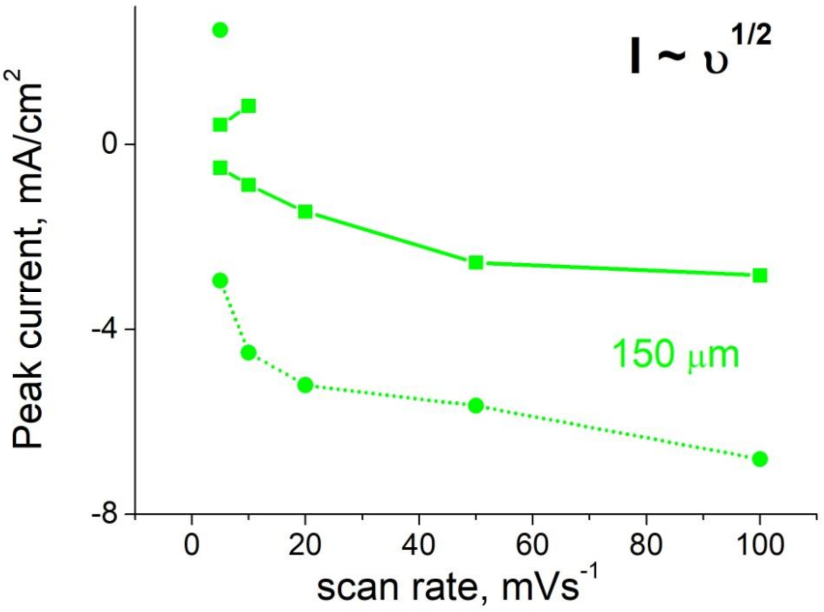


**Figure S5l.** Battery-type (square root) scan rate dependence of the peak current for 150 μm thick por-Si samples with (dotted curve) and without (solid curve) GLC.

**Section S6. EIS**

Nyquist plots (inset: high frequency detail plots) for the different samples before (contour squares) and after (filled squares) GLC deposition.

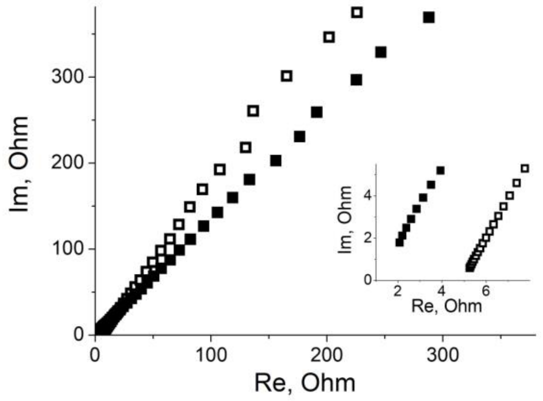


**Figure S6a**. 13 μm thick sample.

The starting point in the high frequency region: 5 Ω vs 2 Ω for por-Si and por-Si+GLC, respectively.

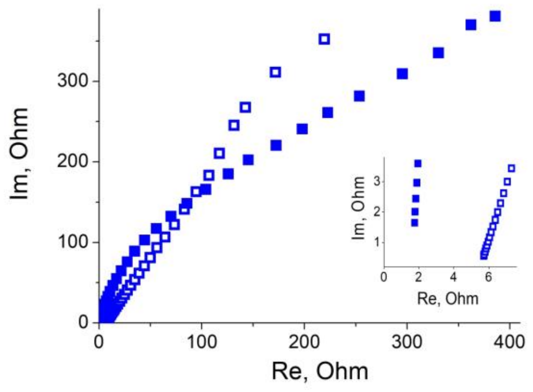


**Figure S6b**. 19 μm thick sample.

The starting point in the high frequency region: 5.7 Ω vs 2 Ω for por-Si and por-Si+GLC, respectively.

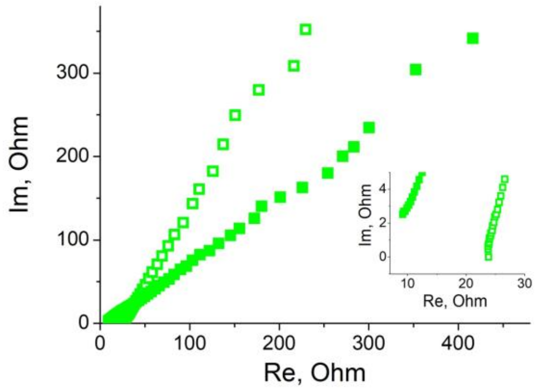


**Figure S6c**. 150 μm thick sample.

The starting point in the high frequency region: 9 Ω vs 24 Ω for por-Si and por-Si+GLC, respectively.

**Section S7. Gravimetric capacitance**

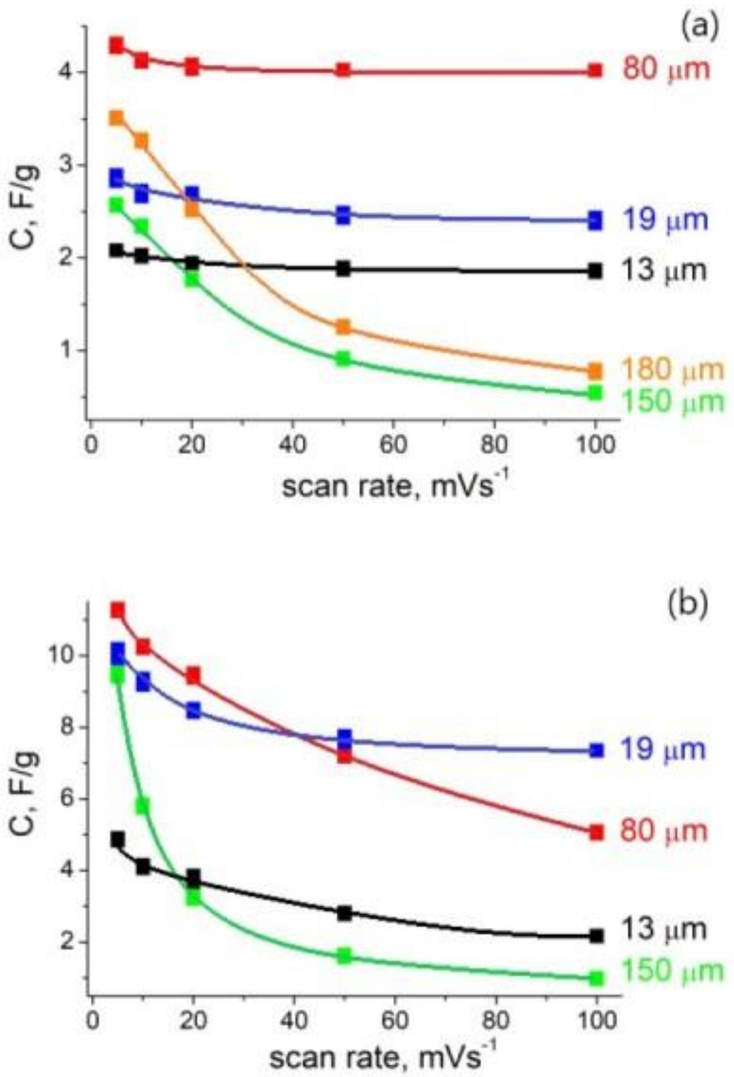


**Figure S7.** Scan rate dependence of the gravimetric capacity at different depth of porous layer for the por-Si samples before (**a**) and after (**b**) GLC synthesis.

**Section S8. GCD data**

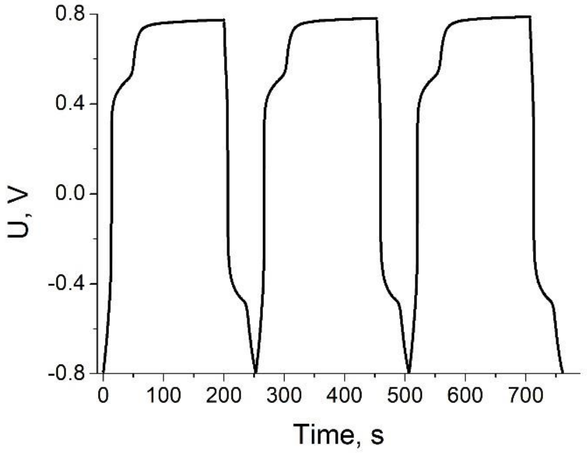


**Figure S8a**. GCD profile at 0.785 mA for 80 μm thick porous layer before GLC synthesis.

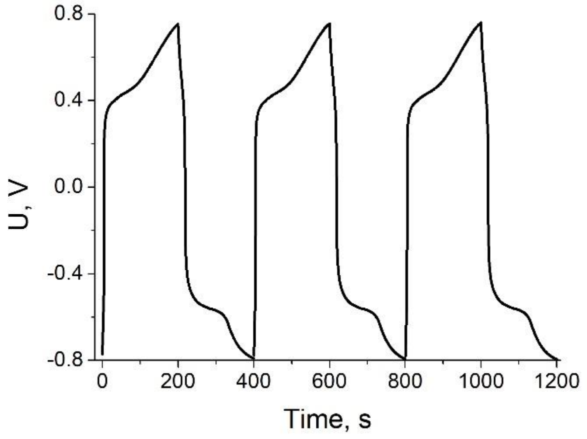


**Figure S8b**. GCD profile at 0.785 mA for 80 μm thick porous layer after GLC synthesis.

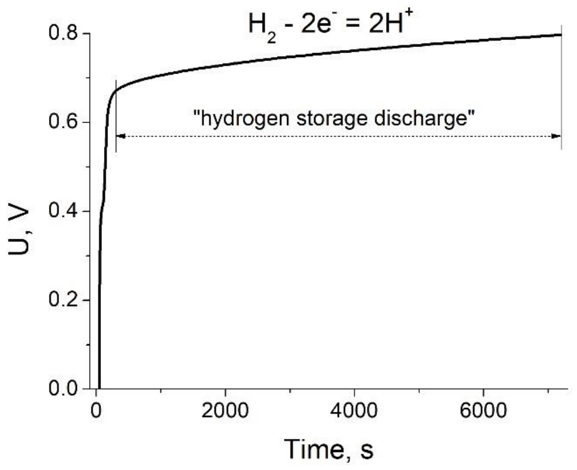


**Figure S8c**. GCD profile at 0.2 mA for 150 μm thick porous layer before GLC synthesis.

**Section S9. Area calculations from reported data**

In reference 13, 17, 19 from main text were not reported the area-normalized capacity data. However, the mentioned paper contained all the information necessary for the calculation.

Citing ref. 13 and 17, "Approximately 6 μm thick porous layer was prepared…". The area-normalized capacity values can be obtained from the volume-normalized ones through simply dividing by the porous layer thickness:

$$7.3 \mathrm{F/cm^3} \times (6 \times 10^{-4}\ \mathrm{cm}) = 4.38 \times 10^{-3} \mathrm{F/cm^2} = 4.38\ \mathrm{mF/cm^2}$$

$$15\ \mathrm{F/cm^3} \times (6 \times 10^{-4}\ \mathrm{cm}) = 9 \times 10^{-3} \mathrm{F/cm^2} = 9\ \mathrm{mF/cm^2}$$

In Romanitan's work (ref. 19) data on the active mass are provided in *Materials characterization* section: "the active mass of NC_ J electrode was calculated to be 90.8 μg while the NC_CV was found to be 74.2 μg." An active area (equal to 0.49 $cm^2$) is given in Table S5. So, area-normalized capacitances were calculated as follows:

$$142\ \mathrm{F/g} = 142 \times (90.8 \times 10^{-6}\ \mathrm{g}) \times \left(\frac{1}{0.49\ \mathrm{cm^2}}\right) = 26.31\ \mathrm{mF/cm^2}$$

$$83\ \mathrm{F/g} = 142 \times (74.2 \times 10^{-6}\ \mathrm{g}) \times \left(\frac{1}{0.49\ \mathrm{cm^2}}\right) = 15.38\ \mathrm{mF/cm^2}$$
